# An Integrated Pathophysiological and Clinical Perspective of the Synergistic Effects of Obesity, Hypertension, and Hyperlipidemia on Cardiovascular Health: A Systematic Review

**DOI:** 10.7759/cureus.72443

**Published:** 2024-10-26

**Authors:** Pedro Okoh, Damilare A Olusanya, Okechukwu C Erinne, Kosisochi E Achara, Abiodun O Aboaba, Rejoice Abiodun, Grace A Gbigbi-Jackson, Rejoice F Abiodun, Adebimpe Oredugba, Ron Dieba, Okelue E Okobi

**Affiliations:** 1 Emergency Medicine, Lancashire Teaching Hospital, Preston, GBR; 2 General Medicine, West Suffolk Hospital, Bury Saint Edmunds, GBR; 3 Epidemiology, University of Texas Health Science Center at Houston, Houston, USA; 4 Public Health, Emory University, Georgia, USA; 5 Family and Community Medicine, Avalon University School of Medicine, Madisonville, USA; 6 Department of Obstetrics and Gynecology, St. Ann’s Bay Regional Hospital, St. Ann’s Bay, JAM; 7 Family Medicine, All Saints University School of Medicine, Roseau, DMA; 8 Internal Medicine, Spartan Health Sciences University, Vieux Fort, JAM; 9 Internal Medicine, Lister Hospital, East and North Hertfordshire NHS Trust, Hertfordshire, GBR; 10 Family Medicine, International University of the Health Sciences, Toronto, CAN; 11 Family Medicine, Medficient Health Systems, Laurel, USA; 12 Family Medicine, Lakeside Medical Center, Belle Glade, USA; 13 Family Medicine, Larkin Community Hospital Palm Springs Campus, Miami, USA

**Keywords:** cardiovascular health, hyperlipidemia, hypertension, insulin resistance, obesity

## Abstract

This review paper explores the synergistic effects of obesity, hypertension (HTN), and hyperlipidemia on cardiovascular health by integrating pathophysiological and clinical perspectives. Obesity, characterized by excessive body fat, HTN, defined by elevated blood pressure, and hyperlipidemia, indicated by high blood lipid levels, are globally prevalent conditions that significantly increase the risk of cardiovascular diseases (CVDs). The interplay between these conditions exacerbates cardiovascular risk through mechanisms such as chronic inflammation, insulin resistance, endothelial dysfunction, arterial stiffness, and atherogenesis. This review synthesizes epidemiological evidence and highlights the prevalence and co-occurrence of these conditions, with an emphasis on their combined impact on cardiovascular health. The literature search encompassed various databases, and data extraction included key study characteristics and outcomes. The findings underscore the importance of integrated management strategies, involving lifestyle interventions, pharmacological treatments, and regular monitoring, to mitigate the heightened cardiovascular risk posed by these conditions. In addition, the various public health implications are addressed, advocating for community-based interventions and policy changes. Future research directions may include exploring novel therapeutic approaches, personalized medicine strategies, and longitudinal studies to enhance the understanding and management of the synergistic effects of obesity, HTN, and hyperlipidemia on cardiovascular health.

## Introduction and background

Obesity, hypertension (HTN), and hyperlipidemia are three interconnected conditions with significant global prevalence and implications for public health [1-3]. Obesity is characterized by an excessive accumulation of body fat, typically defined by a body mass index (BMI) of 30 or higher [1-4]. The World Health Organization (WHO) estimates that more than 650 million adults worldwide were obese in 2016, with the prevalence rising rapidly across various regions, particularly in low- and middle-income countries [5]. Furthermore, it is noteworthy that, globally, while obesity prevalence has over doubled since 1990, adolescent obesity prevalence has quadrupled. Consequently, as of 2022, 2.5 billion adults (43%) aged 18 years and above were overweight, with 890 million (16%) of them being obese, even as 35 million children aged below five years were overweight [5]. Moreover, in 2022, more than 390 million children and adolescents aged between five and 19 years were overweight, with over 160 million of them being obese [5]. HTN, often termed the “silent killer,” is defined by consistently elevated blood pressure levels, specifically a systolic blood pressure of 130 mm Hg or higher and/or a diastolic blood pressure of 80 mm Hg or higher. HTN affects over 1.3 billion people globally, with significant variability in prevalence among different populations and regions [1]. Hyperlipidemia, characterized by elevated levels of lipids in the blood, including cholesterol and triglycerides, is a major risk factor for cardiovascular diseases (CVDs) [6]. The prevalence of hyperlipidemia varies widely but is notably high in populations with significant dietary and lifestyle risk factors [6]. In their study, Esteghamati et al. reported an obesity, HTN, and dyslipidemia prevalence of 22.3%, 25.2%, and 42.9%, respectively, highlighting the considerable burden of these conditions [2].

The public health significance of obesity, HTN, and hyperlipidemia cannot be overstated, given their roles as primary risk factors for CVD, the leading cause of death worldwide [1-3]. The growing epidemic of these conditions is driven by a combination of genetic, behavioral, and environmental factors [2]. Urbanization, sedentary lifestyles, and the global adoption of energy-dense, nutrient-poor diets contribute significantly to the rising prevalence of obesity, which in turn exacerbates the rates of HTN and hyperlipidemia [6]. Bozkurt et al. emphasize that the interplay between these conditions exacerbates the overall cardiovascular risk, creating a synergistic effect that amplifies morbidity and mortality [3]. The increasing prevalence of these conditions, particularly in younger age groups, poses a significant challenge for health systems worldwide, requiring comprehensive public health strategies to address lifestyle modifications, early diagnosis, and effective management.

This review explores the synergistic effects of obesity, HTN, and hyperlipidemia on cardiovascular health, providing a comprehensive understanding of how these conditions interact and amplify each other's impact. The pathophysiological mechanisms underlying this synergy involve complex interactions between metabolic, hormonal, and inflammatory pathways [7]. For instance, obesity-induced insulin resistance can lead to HTN through mechanisms such as increased sympathetic nervous system activity and sodium retention [2-4]. Concurrently, obesity contributes to hyperlipidemia by altering lipid metabolism, increasing triglycerides, and reducing high-density lipoprotein (HDL) cholesterol levels [2-4]. Wang et al. highlight that these interrelated processes can synergistically increase the risk of ischemic stroke, underscoring the importance of understanding these interactions for effective clinical management [4]. In addition to elucidating the pathophysiological mechanisms, this review will discuss epidemiological evidence highlighting the prevalence and co-occurrence of these conditions and their combined impact on cardiovascular health. Mohamed suggests that addressing these conditions through functional foods and dietary interventions can be a promising strategy, further highlighting the need for integrated approaches in management [8]. Epidemiological studies provide critical insights into the extent of the problem and identify key demographic and lifestyle factors contributing to the observed trends. This review will synthesize findings from various population-based studies to present a clear picture of the public health burden posed by these interrelated conditions.

## Review

Materials and methods: search strategy, data extraction, and synthesis

The literature search was conducted across several comprehensive databases, including the American Heart Association (AHA), PubMed, NIH, Cochrane Library, BMC, Frontiers, Oxford Academic, MDPI, and Elsevier, to gather relevant studies examining the interrelationship between obesity, HTN, and hyperlipidemia on cardiovascular health. Keywords used in the search strategy included “obesity,” “hypertension,” “hyperlipidemia,” and “cardiovascular health.” These terms were used both individually and in combination to ensure a broad and inclusive search of the existing literature. The review included cohort studies, randomized controlled trials (RCTs), and observational studies investigating the synergistic effects of obesity, HTN, and hyperlipidemia on cardiovascular health. The criteria for exclusion included non-human studies, non-English articles, studies without full text available, case reports, reviews, and editorials. In addition, studies with sample sizes of fewer than 50 participants, those with poor methodological quality, and studies published before 2000 were excluded to ensure the relevance and reliability of the included data. Data extraction included systematically examining each selected study to extract critical information about the synergistic effects of obesity, HTN, and hyperlipidemia on cardiovascular health. Key data points collected encompassed study characteristics (e.g., author(s), publication year, study design), participant characteristics (e.g., sample size, demographics), intervention details, and primary outcomes related to the interactions between obesity, HTN, and hyperlipidemia. The extraction process also involved documenting the statistical methods used and the results obtained, including association and effect size measures. Quality assessment was performed independently by two reviewers using standardized assessment tools to ensure the reliability and validity of the included studies. Randomized controlled trials were evaluated using the Cochrane Risk of Bias tool, which assesses various bias domains such as selection, performance, detection, attrition, and reporting biases. The extracted data were synthesized using qualitative methods. Qualitative synthesis involved thematic analysis to identify and examine recurrent themes and patterns across the studies, providing a comprehensive understanding of the synergistic effects of obesity, HTN, and hyperlipidemia on cardiovascular health. The synthesis provides a detailed understanding of the interactions and combined effects of obesity, HTN, and hyperlipidemia on cardiovascular health [9-11].

**Figure 1:**
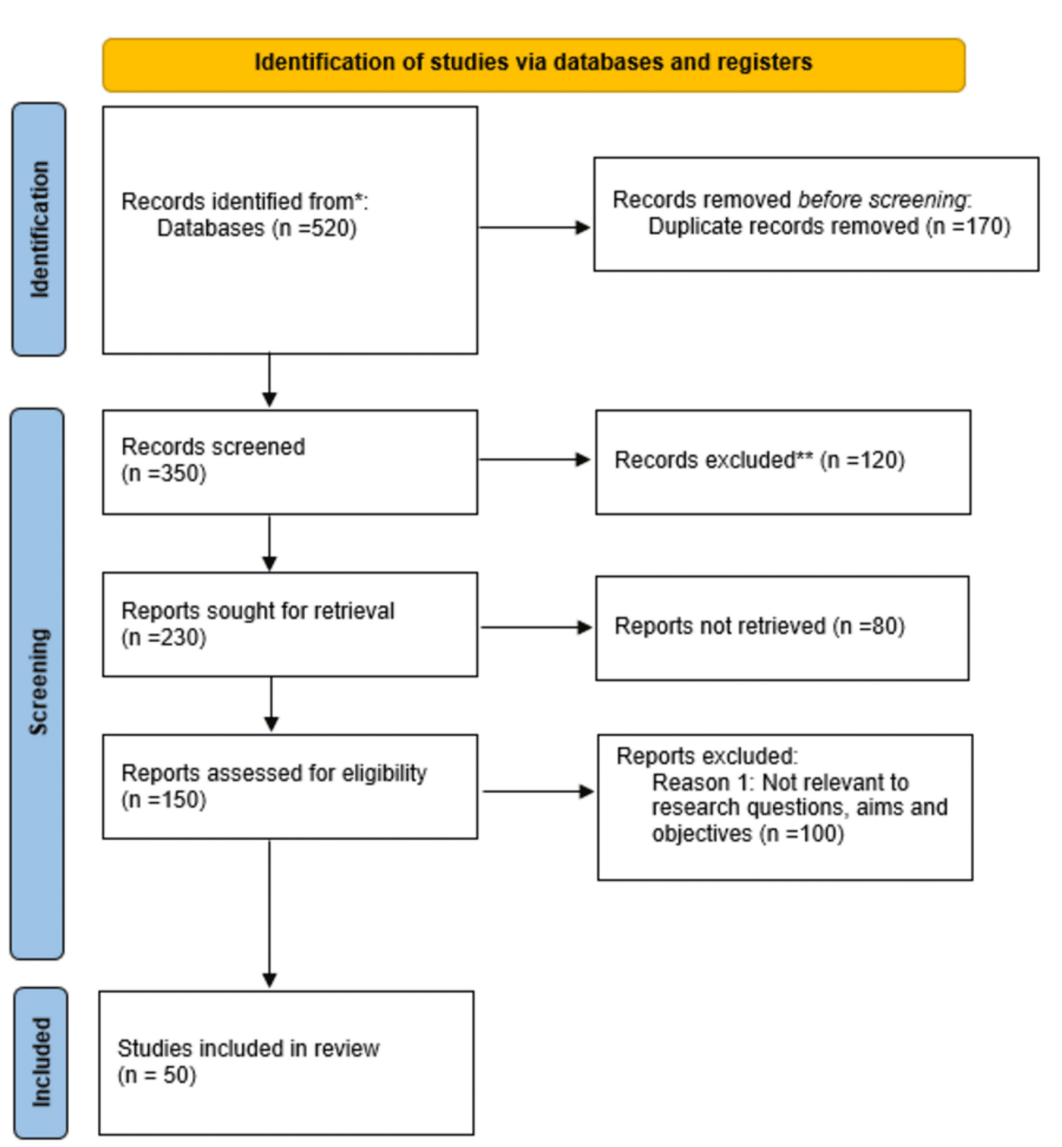
PRISMA flow diagram illustrating the study selection process for this review

The in-depth search conducted on the various online databases led to the identification of a total of 500 studies. After removing the duplicates and screening titles and abstracts, 150 studies were assessed for eligibility based on full-text review. Finally, 50 studies met the inclusion criteria and were included in the systematic review. The study selection process for this review is presented in Figure 1.

The results of the Cochrane Risk of Bias tool analysis are presented in Table 2.

**Table 1:** Presentation of the Cochrane Risk of Bias tool analysis outcomes

Study	Bias domain	Judgment	Support for judgment
Mills KT, Stefanescu A, He J, 2020 [1]	Random sequence generation (selection bias)	Low	This is a narrative review and does not involve random sequence generation.
Esteghamati A, Meysamie A, Khalilzadeh O, et al., 2009 [2]	Random sequence generation (selection bias)	Low	The study is based on a national survey with appropriate random sampling.
Bozkurt B, Aguilar D, Deswal A, et al., 2016 [3]	Selection bias	High	There is an increased probability of the selection of evidence being influenced by expert opinion as opposed to systematic methods, as this is a scientific statement and not a study.
Wang C, Du Z, Ye N, et al., 2022 [4]	Selection bias	Moderate	Observational study; potential biases in participant selection.
Mohamed S, 2014 [5]	Reporting bias	High	Potential for selective reporting based on the author’s focus, alongside the potential for bias from the interpretation of the evidence.
Tawfik GM, Dila KAS, Mohamed MYF, et al., 2019 [6]	Reporting bias	Low	Results are clearly reported, and all expected outcomes are addressed.
Gurevitch J, Koricheva J, Nakagawa S, Stewart G, 2018 [7]	Selection bias	Low	The study provides a comprehensive overview of meta-analysis techniques, ensuring systematic selection and assessment.
Batten J, Brackett A, 2021 [8]	Attrition bias	Low	The study covers all necessary steps, reducing the risk of missing data.
Theofilis P, Sagris M, Oikonomou E, et al., 2021 [9]	Performance bias	Moderate	The study's narrow focus may reduce performance bias, but specifics are not fully detailed.
Wu H, Ballantyne CM, 2020 [10]	Selection bias	High	The study’s selection criteria for included research are not fully transparent.
Hill MA et al., 2021 [11]	Selection, attrition, and reporting biases	Low	All potential confounding factors have been fully and aptly addressed.
Boutouyrie P et al., 2021 [12]	Reporting bias	Moderate	All possible confounding factors have not been fully addressed, particularly with regard to the funding disclosures.
Chirinos JA et al., 2019 [13]	Reporting bias	Low	All potential confounding factors have been fully and aptly addressed.
Alsharari R et al., 2019 [14]	Selection and detection bias	Moderate	The selection of participants may introduce bias. The blinding has not been well described.
Khosravi M et al., 2019 [15]	Selection and other bias	Moderate	Potential confounding factors, including the complexity of oxidative stress markers
Petrucci G et al., 2022 [16]	Other biases	Low	Potential conflicts of interest
Lechner K et al., 2019 [17]	Attrition bias	Low	The study covers all necessary steps, reducing the risk of missing data.
Caiati C et al., 2023 [18]	Other biases	Low	There is an increased potential for publication bias.
St. Paul A et al., 2020 [19]	Selection bias	Low	The study provides a comprehensive overview of meta-analysis techniques, ensuring systematic selection and assessment.
Ji Q et al., 2024 [20]	Selection bias	Moderate	There is a possibility of the retrospective, cross-sectional design introducing selection bias and other confounding factors. Nonetheless, the bigger multicenter dataset reduces the bias.
Jain RB, 2020 [21]	Reporting bias	High	Potential selection bias resulting from the study’s observational nature. Restricted control over the confounding factors along with the potential issues related to the reliability of exposure measurement.
Cleven L et al., 2020 [22]	Selection bias	Low	The systematic review has a rigorous methodology despite the potential risk of publication bias.
Chandrabose et al., 2018 [23]	Detection bias	Low	Systematic review and meta-analysis have some robust study selection criteria despite the potential risk from study heterogeneity, even though this has been well-controlled.
Dayimu et al., 2019 [24]	Performance bias	Moderate	The longitudinal cohort study design has reduced the potential biases, even though there is potential for attrition bias and various confounding variables.
Torky et al., 2021 [25]	Selection bias	Low	Longitudinal design strengthens causal inference but may still have issues with attrition and unmeasured confounding.
Abera et al., 2019 [26]	Performance bias	High	Single-center study in a specific population (Ethiopia), with potential selection bias and limited generalizability. Lack of randomization and possible confounders contribute to a higher risk.
Bitencourt et al., 2023 [27]	Selection bias	Moderate	Cohort design reduces some bias, but possible issues with the accuracy of self-reported data and potential for residual confounding.
Clark et al., 2019 [28]	Selection bias	Low	Large, population-based cohort study with rigorous methods reduces some bias. Some residual confounding might exist, but the overall risk is low.
Lindh et al., 2019 [29]	Attrition bias	Moderate	Population-based register data provides robustness but potential misclassification bias and residual confounding are possible.
Alieva et al., 2020 [30]	Reporting bias	Moderate	Data from two population-based studies enhances reliability, but the risk of bias from study heterogeneity and unmeasured confounders is moderate.
Irawati et al., 2018 [31]	Performance bias	Moderate	The blinding of the study participants is not clear.
Rabi DM, et al., 2020 [32]	Selection bias	Moderate	The guidelines used were mainly based on the available evidence.
Litwin M, Kułaga Z, 2021 [33]	Performance bias	Moderate	The study has increased variability with regard to clinical practices.
Elkins C, et al., 2019 [34]	Selection bias	Moderate	The evidence for the guidelines used is limited.
Alonso R, et al., 2020 [35]	Selection bias	Moderate	Increased variability has been observed in the diagnosis and treatment approaches.
Christian Flemming GM, et al., 2020 [36]	Detection bias	Moderate	There were variations in the definition of metabolic syndrome.
Belardo D, et al., 2022 [37]	Selection bias	low	The study made use of evidence-based approaches.
Barone Gibbs B, et al., 2021 [38]	Selection bias	Low	A scientific statement that is based on a larger body of evidence was used.
Rippe JM, 2019 [39]	Selection bias	Low	The study presented a comprehensive lifestyle review.
Carey RM, et al., 2022 [40]	Selection bias	Low	The study presented an evidence-based review.
Korhonen MJ, et al. (2020) [41]	Detection bias	Low	The study has utilized self-reported lifestyle changes, which increases the risk of false data.
Lip GYH, Lane DA, Lenarczyk R, et al., 2022 [42]	Selection bias	Moderate	Limited information regarding the randomization
Buckley BJR, Lip GYH, 2022 [43]	Selection bias	Moderate	Limited information on the participant selection process
Giallauria F, Strisciuglio T, Cuomo G, et al., 2021 [44]	Detection bias	Moderate	There is an increased possibility for subjective outcome measures.
Commodore-Mensah Y, Loustalot F, Himmelfarb CD, et al., 2020 [45]	Performance bias	Low	The study has utilized standardized workshop protocols.
Roger VL, Sidney S, Fairchild AL, et al., 2020 [46]	Selection bias	Moderate	Policy statements presented have limited participant data.
Mendis S, Graham I, Narula J, 2022 [47]	Selection bias	Moderate	More focus has been placed on scalable frameworks, which increases the potential selection bias.
Hirashiki A, Shimizu A, Nomoto K, et al., 2022 [48]	Selection bias	Low	Limited selection detail for community interventions
Ndejjo R, Hassen HY, Wanyenze RK, et al., 2021 [49]	Attrition bias	Moderate	Increased risk of dropout among participants
Soltani S, Saraf-Bank S, Basirat R, et al., 2021 [50]	Selection bias	Moderate	Increased variability in the community-based study selection

Discussion

Pathophysiological Mechanisms

Obesity significantly impacts cardiovascular health through various pathophysiological mechanisms, including chronic inflammation, insulin resistance, and endothelial dysfunction. Obesity is characterized by an excessive accumulation of adipose tissue, which secretes a range of pro-inflammatory cytokines such as tumor necrosis factor-alpha (TNF-α) and interleukin-6 (IL-6). These cytokines contribute to systemic inflammation and endothelial dysfunction, a critical factor in the development of atherosclerosis and cardiovascular disease [12]. In addition, obesity is closely linked to insulin resistance, a condition where cells fail to respond effectively to insulin, leading to elevated blood glucose levels. Hyperglycemia, which is due to insulin resistance, exacerbates oxidative stress by increasing reactive oxygen species (ROS) production within the endothelial cells [13]. Normally, oxidative stress damages the endothelial linings, which, in turn, worsens vascular dysfunction while simultaneously promoting atherosclerosis [13]

Insulin resistance further exacerbates cardiovascular risk by promoting dyslipidemia and HTN. Insulin-resistant states are associated with increased free fatty acid flux, contributing to lipid abnormalities and endothelial cell damage [10]. Furthermore, obesity-induced insulin resistance increases sympathetic nervous system activity and sodium retention, which elevates blood pressure [14]. The combination of these factors creates a vicious cycle where obesity, inflammation, and metabolic disturbances collectively enhance cardiovascular risk.

Obesity and Cardiovascular Health: HTN, Hyperlipidemia, and Cardiovascular Health

HTN adversely affects cardiovascular health through several pathophysiological pathways, notably arterial stiffness and left ventricular hypertrophy. Arterial stiffness, characterized by the reduced elasticity of large arteries, is a hallmark of HTN and contributes to increased cardiac workload and pressure. This condition is driven by structural changes in the arterial wall, including increased collagen deposition and reduced elastin content, which are often exacerbated by chronic high blood pressure [15]. The stiffening of arteries results in elevated systolic blood pressure and pulse pressure, promoting further cardiovascular damage. Left ventricular hypertrophy (LVH), another common consequence of HTN, involves the thickening of the heart's left ventricular wall as a compensatory response to increased vascular resistance and pressure overload. LVH is associated with impaired diastolic function, increased myocardial oxygen demand, and a higher risk of arrhythmias, all of which significantly elevate the risk of heart failure and other cardiovascular events [16]. Furthermore, endothelial dysfunction and increased oxidative stress, common in hypertensive individuals, exacerbate arterial damage and contribute to atherosclerosis development [17].

Hyperlipidemia plays a critical role in the pathogenesis of atherosclerosis and subsequent cardiovascular events through mechanisms such as plaque formation and oxidative stress. Elevated levels of low-density lipoprotein cholesterol (LDL-C) are particularly harmful, as LDL-C particles infiltrate the endothelial lining of arteries, becoming oxidized and initiating inflammatory processes that lead to plaque formation. The accumulation of lipid-laden macrophages, or foam cells, within the arterial wall is a key step in atherogenesis, eventually resulting in the development of fatty streaks and fibrous plaques [18]. Oxidative stress further contributes to the instability of these plaques by promoting the oxidation of LDL-C and the production of reactive oxygen species (ROS), which damage endothelial cells and smooth muscle cells within the vessel wall [18]. This oxidative damage not only accelerates plaque growth but also increases the risk of plaque rupture and thrombosis, leading to acute cardiovascular events such as myocardial infarction and stroke [19]. Hyperlipidemia-induced endothelial dysfunction, characterized by impaired nitric oxide bioavailability and increased endothelial cell permeability, also exacerbates the atherogenic process [20].

Synergistic Effects of Obesity, HTN, and Hyperlipidemia on Cardiovascular Health

**Figure 2:**
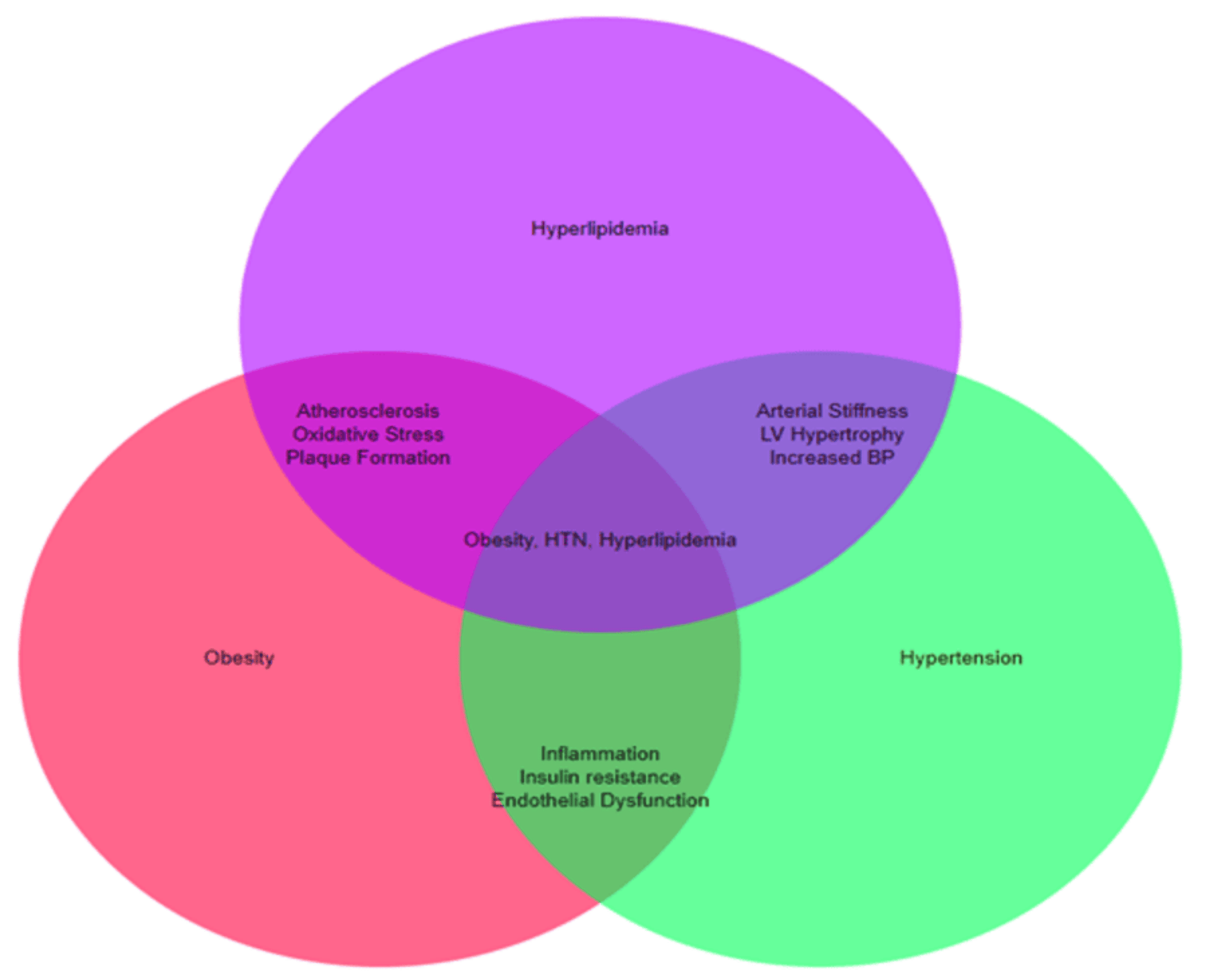
Synergistic pathophysiological links between obesity, hypertension, and hyperlipidemia on cardiovascular health. Image Credits: Okelue E. Okobi (corresponding author)

The combined effects of obesity, HTN, and hyperlipidemia on cardiovascular health are substantial and additive, leading to compounded pathophysiological processes that significantly elevate cardiovascular risk. Each condition independently contributes to cardiovascular disease, but their co-occurrence creates a complex interplay that amplifies their individual effects. For instance, obesity exacerbates HTN through mechanisms such as increased sympathetic nervous system activity and insulin resistance, while hyperlipidemia accelerates atherogenesis by promoting lipid accumulation and oxidative stress. The presence of all three conditions leads to a heightened inflammatory state, with increased levels of pro-inflammatory cytokines and oxidative stress markers [21]. This inflammatory milieu contributes to endothelial dysfunction, arterial stiffness, and the progression of atherosclerosis. In addition, the metabolic disturbances associated with obesity and insulin resistance promote dyslipidemia and HTN, creating a vicious cycle that perpetuates cardiovascular damage [22]. The synergistic interaction between these conditions significantly increases the likelihood of adverse cardiovascular events, including myocardial infarction, stroke, and heart failure, underscoring the importance of integrated management strategies to address this triad of risk factors. The synergistic pathophysiological links between obesity, HTN, and hyperlipidemia are presented in Figure 2.

Epidemiological Evidence: Prevalence and Co-occurrence

The global prevalence of obesity, HTN, and hyperlipidemia is alarmingly high, reflecting significant public health challenges. According to recent estimates, over 1.9 billion adults were overweight, with more than 650 million classified as obese globally. HTN affects approximately 1.13 billion people worldwide, with the highest prevalence in low- and middle-income countries [1,5]. Hyperlipidemia, characterized by elevated levels of cholesterol and triglycerides, is a prevalent condition associated with an increased risk of cardiovascular diseases. In China, a large-scale cross-sectional study reported a co-prevalence of these conditions among adults with type 2 diabetes mellitus, highlighting the compounded risk they pose [22,23]. In the United States, Jain noted a significant overlap of obesity with conditions like diabetes, anemia, and HTN, further complicating management and treatment outcomes [21]. The co-occurrence of these conditions not only increases the individual risk of cardiovascular events but also poses a significant burden on healthcare systems globally [23-25]. Regional variations exist, with developing countries experiencing rapid increases due to urbanization and lifestyle changes. By contrast, developed countries continue to struggle with high prevalence rates due to persistent dietary and physical activity patterns.

Longitudinal Studies

Longitudinal studies have provided valuable insights into the combined impact of obesity, HTN, and hyperlipidemia on cardiovascular health. Cleven et al. conducted a systematic review of longitudinal studies and found a strong association between physical inactivity and the incidence of obesity, coronary heart disease, diabetes, and HTN [25]. These findings underscore the importance of lifestyle factors in the progression of these conditions and their combined effect on cardiovascular health. Chandrabose et al. highlighted the role of the built environment in influencing cardio-metabolic health through a meta-analysis of longitudinal studies, emphasizing how urban planning and access to recreational spaces can mitigate the risk factors associated with obesity, HTN, and hyperlipidemia [26]. Another significant study by Dayimu et al. examined lipid profile trajectories and their relationship with cardiovascular disease risk, revealing that persistent dyslipidemia significantly increases the risk of cardiovascular events over time [27]. Torky et al. focused on individuals with congenital adrenal hyperplasia. They found that longitudinal monitoring of cardiovascular risk factors, including obesity, HTN, and hyperlipidemia, is crucial for early intervention and management [28].

Population Studies

The key population-based studies that have highlighted the increased risk of cardiovascular events in persons with obesity, HTN, and hyperlipidemia have been presented in Table 2. 

**Table 2:** Key population-based studies highlighting the increased risk of cardiovascular events in individuals with obesity, hypertension (HTN), and hyperlipidemia

Study	Population	Key findings
Abera et al., 2019 [29]	Diabetic patients in Ethiopia	High prevalence of HTN and dyslipidemia among diabetic patients; significant association with increased cardiovascular risk.
Ji et al., 2024 [23]	Chinese adults with type 2 diabetes	High co-prevalence of obesity, HTN, and hyperlipidemia; increased risk of cardiovascular events.
Jain, 2020 [24]	U.S. population	Co-occurrence of obesity with diabetes, anemia, HTN; elevated concentrations of perfluoroalkyl acids linked to cardiovascular risk.
Bitencourt et al., 2023 [30]	Global diabetic population	Co-occurrence of periodontitis and diabetes-related complications; increased cardiovascular morbidity.
Clark et al., 2019 [31]	Black adults in the U.S.	Hypertension attributed to a significant proportion of cardiovascular disease risk; need for targeted interventions.
Lindh et al., 2019 [32]	Swedish population	High cardiovascular event rates in individuals with multiple atherosclerotic risk factors, including obesity and HTN.
Alieva et al., 2020 [33]	Russian and European populations	High prevalence of metabolic syndrome and its strong association with cardiovascular disease risk.
Irawati et al., 2019 [34]	Asian populations	Long-term incidence of cardiovascular events; significant risk factors include obesity, HTN, and hyperlipidemia.

Clinical Implications: Diagnosis and Screening

Screening and early diagnosis of obesity, HTN, and hyperlipidemia are crucial for preventing cardiovascular diseases and managing these conditions effectively. According to the Canadian Journal of Cardiology, routine measurement of body mass index (BMI) and waist circumference is recommended for identifying obesity, as these metrics provide insights into overall and central adiposity, respectively [34]. Early diagnosis of HTN involves regular blood pressure monitoring, with thresholds for normal and elevated blood pressure well-defined in clinical guidelines. Home and ambulatory blood pressure monitoring is also recommended to obtain accurate measurements and detect white-coat HTN [35,36]. For hyperlipidemia, lipid profile assessments, including total cholesterol, low-density lipoprotein cholesterol (LDL-C), high-density lipoprotein cholesterol (HDL-C), and triglycerides, should be conducted regularly, especially in individuals with risk factors such as obesity and HTN [37]. The importance of screening for familial hypercholesterolemia, a genetic disorder that significantly elevates cholesterol levels, is highlighted by Alonso et al., who emphasize early intervention to prevent severe cardiovascular outcomes [38]. In addition, integrating metabolic syndrome criteria, which include measures of obesity, blood pressure, and lipid levels, can help identify individuals at high risk for cardiovascular diseases [39]. Early diagnosis and consistent monitoring are essential for timely intervention and effective management of these interrelated conditions.

Management Strategies

Effective management of obesity, HTN, and hyperlipidemia involves significant lifestyle modifications. Dietary changes, such as adopting a heart-healthy diet rich in fruits, vegetables, whole grains, lean proteins, and healthy fats, play a crucial role in managing these conditions [40]. Physical activity is another critical component, with the American Heart Association recommending at least 150 minutes of moderate-intensity exercise per week to improve cardiovascular health and aid in weight management [41]. Weight management programs that combine dietary changes, physical activity, and behavioral modifications effectively reduce obesity and associated cardiovascular risks [42].

Lifestyle Interventions, Pharmacological Treatments, and Integrated Approach

Pharmacological treatments are often necessary for managing HTN and hyperlipidemia, particularly when lifestyle modifications alone are insufficient. Antihypertensives, including ACE inhibitors, ARBs, beta-blockers, calcium channel blockers, and diuretics, are commonly prescribed to control blood pressure and reduce the risk of cardiovascular events [43]. For hyperlipidemia, statins are the primary treatment to lower LDL-C levels and prevent atherosclerotic cardiovascular disease. In cases of combined dyslipidemia, additional lipid-lowering agents such as ezetimibe, PCSK9 inhibitors, and fibrates may be used [44]. Moreover, pharmacological treatments that include GLP-1 receptor agonists such as liraglutide and semaglutide have indicated effectiveness in attaining considerable weight loss and enhancing cardiovascular outcomes [44-46]. Such pharmacological treatments promote weight loss while also offering favorable effects with regard to blood pressure and glycemic control, which are considered vital to the management of hyperlipidemia and HTN. Furthermore, in instances of severe obesity, in which weight loss is unattainable through pharmacotherapy and lifestyle interventions, bariatric surgery has been used as an effective treatment for weight management [45]. Such surgical interventions often result in sustained weight loss, enhancement in various metabolic parameters, and considerable cardiovascular risk reduction. Further, an integrated approach that combines pharmacological treatments and lifestyle modifications, and when necessary, surgical interventions, is important in tackling the various synergistic effects of HTN, hyperlipidemia, and obesity on cardiovascular health [44, 45]. The coordination of such treatments enables healthcare providers to realize better long-term outcomes while reducing cardiovascular risk events in high-risk populations [45]. Thus, combination therapies are often required for patients with multiple risk factors to achieve optimal cardiovascular protection. This approach involves coordinated care that addresses all three conditions simultaneously through lifestyle interventions, pharmacological treatments, and regular monitoring. Integrated care models emphasize the importance of patient education, self-management, and multidisciplinary collaboration among healthcare providers [45]. Comprehensive cardiovascular rehabilitation programs that include exercise training, nutritional counseling, and psychosocial support have been shown to improve outcomes and reduce the recurrence of cardiovascular events [46]. Healthcare providers can ensure that all aspects of a patient's cardiovascular risk are addressed, leading to better long-term health outcomes and reduced healthcare costs [47].

Public Health Implications

Public health initiatives aimed at preventing obesity, HTN, and hyperlipidemia are crucial to mitigating the burden of cardiovascular diseases globally. Programs such as community health education campaigns, school-based interventions, and workplace wellness programs are essential in promoting healthy lifestyles. These initiatives focus on encouraging physical activity, balanced diets, and regular health screenings to detect and manage these conditions early [25]. Integrating these programs into primary healthcare services can ensure broader reach and sustainability, particularly in underserved communities. For example, initiatives like the Million Hearts campaign in the United States aim to prevent one million heart attacks and strokes within five years by implementing evidence-based interventions to improve cardiovascular health [48]. Moreover, addressing social determinants of health, such as access to healthy food and safe spaces for physical activity, is fundamental to these prevention strategies. 

Prevention Strategies, Policy Recommendations, and Community and Environmental Interventions

To address the combined burden of obesity, HTN, and hyperlipidemia on public health systems, comprehensive policy changes are necessary. Policies that promote healthy eating through subsidies for fruits and vegetables, taxation of sugary drinks, and regulation of food marketing to children can significantly impact dietary habits [49]. In addition, implementing policies that encourage physical activity, such as developing pedestrian-friendly infrastructure and public recreational spaces, can help reduce the prevalence of these conditions [50]. Healthcare policies should also support regular screening and management of obesity, HTN, and hyperlipidemia through universal healthcare coverage and preventive care incentives. Strengthening surveillance systems to monitor the incidence and prevalence of these conditions and their risk factors is crucial for informed policy-making and resource allocation [49]. Community-based interventions and environmental modifications play a vital role in preventing and managing obesity, HTN, and hyperlipidemia. Community interventions, such as group-based exercise programs, nutrition workshops, and peer support groups, effectively promote lifestyle changes and improve health outcomes [51]. Environmental modifications, including creating parks, walking trails, and bike lanes, encourage physical activity by providing safe and accessible spaces for exercise [52]. In addition, local policies that support the establishment of farmers' markets and community gardens can improve access to fresh, healthy foods, thereby addressing dietary risk factors for these conditions [53, 54]. Collaborative efforts between public health agencies, local governments, and community organizations are essential to implement these interventions effectively and ensure their sustainability.

Strengths and Limitations

The study leverages its strengths in the comprehensive approach, as it assumes integrated clinical and pathophysiological perspectives, thereby offering a holistic perspective of the way the different factors interact. This provides a more comprehensive understanding of the various cardiovascular health risks. The other notable strength regards the observation that the study and its findings are evidence-based. Thus, the study has utilized an array of evidence sources, including epidemiological studies, cohort studies, systematic reviews, clinical data, and various medical databases, which enables it to offer well-rounded perspectives supported by adequate and diverse evidence. The other notable strength of this study regards cross-disciplinary insights, as the study has integrated findings from divergent studies and fields, which enables it to provide novel insights and interventional strategies. Nevertheless, a number of potential limitations have been acknowledged including the existence of potential for confounding variables. For instance, other notable risk factors, including physical inactivity, genetic predispositions, and smoking, are likely to confound synergistic effects thereby making it increasingly challenging to effectively isolate the contributions attributable to HTN, obesity, and hyperlipidemia. Consequently, the measurement challenges are considered potential limitations in this study, given that the measurement of the combined effects of several risk factors, including HTN, obesity, and hyperlipidemia, in a manner that precisely reflects the real-world clinical results might be difficult. Challenges may be experienced in the separation of correlations from the causations. 

## Conclusions

The synergistic effects of obesity, HTN, and hyperlipidemia significantly elevate the risk of cardiovascular diseases through complex interactions involving chronic inflammation, insulin resistance, endothelial dysfunction, arterial stiffness, and atherogenesis. Individually, each condition poses substantial health risks, but their combined presence amplifies these risks, leading to a higher incidence of myocardial infarction, stroke, and heart failure. This review underscores the importance of understanding these interrelated pathophysiological mechanisms to develop effective prevention and management strategies. Integrated management and prevention strategies are crucial to reducing modifiable cardiovascular risks associated with obesity, HTN, and hyperlipidemia. The coordinated efforts of healthcare providers, policymakers, and communities are essential to mitigate the global burden of these interrelated conditions. Future research should focus on novel therapeutic approaches that address the combined effects of obesity, HTN, and hyperlipidemia. This includes investigating the efficacy of combination therapies and personalized medicine strategies that tailor treatments based on individual risk profiles. Longitudinal studies are needed to evaluate the long-term effectiveness of integrated management strategies and community-based interventions. 

## References

[1] Mills KT, Stefanescu A, He J (2020). The global epidemiology of hypertension. Nat Rev Nephrol.

[2] Esteghamati A, Meysamie A, Khalilzadeh O (2009). Third national surveillance of risk factors of non-communicable diseases (SuRFNCD-2007) in Iran: Methods and results on prevalence of diabetes, hypertension, obesity, central obesity, and dyslipidemia. BMC Public Health.

[3] Bozkurt B, Aguilar D, Deswal A (2016). Contributory risk and management of comorbidities of hypertension, obesity, diabetes mellitus, hyperlipidemia, and metabolic syndrome in chronic heart failure: a scientific statement from the American Heart Association. Circulation.

[4] Wang C, Du Z, Ye N, Shi C, Liu S, Geng D, Sun Y (2022). Hyperlipidemia and hypertension have synergistic interaction on ischemic stroke: insights from a general population survey in China. BMC Cardiovasc Disord.

[5] World Health Organization(WHO), 2024 2024 (2024). Obesity and overweight. https://www.who.int/news-room/fact-sheets/detail/obesity-and-overweight.

[6] Nelson RH (2013). Hyperlipidemia as a risk factor for cardiovascular disease. Prim Care.

[7] DeMarco VG, Aroor AR, Sowers JR (2014). The pathophysiology of hypertension in patients with obesity. Nat Rev Endocrinol.

[8] Mohamed S (2014). Functional foods against metabolic syndrome (obesity, diabetes, hypertension and dyslipidemia) and cardiovasular disease. Trends Food Sci Technol.

[9] Tawfik GM, Dila KA, Mohamed MY, Tam DN, Kien ND, Ahmed AM, Huy NT (2019). A step by step guide for conducting a systematic review and meta-analysis with simulation data. Trop Med Health.

[10] Gurevitch J, Koricheva J, Nakagawa S, Stewart G (2018). Meta-analysis and the science of research synthesis. Nature.

[11] Batten J, Brackett A (2021). Ensuring Rigor in systematic reviews: part 5, quality appraisal, data extraction, synthesis. Heart Lung.

[12] Theofilis P, Sagris M, Oikonomou E, Antonopoulos AS, Siasos G, Tsioufis C, Tousoulis D (2021). Inflammatory mechanisms contributing to endothelial dysfunction. Biomedicines.

[13] Bhatti JS, Sehrawat A, Mishra J (2022). Oxidative stress in the pathophysiology of type 2 diabetes and related complications: current therapeutics strategies and future perspectives. Free Radic Biol Med.

[14] Wu H, Ballantyne CM (2020). Metabolic inflammation and insulin resistance in obesity. Circ Res.

[15] Hill MA, Yang Y, Zhang L, Sun Z, Jia G, Parrish AR, Sowers JR (2021). Insulin resistance, cardiovascular stiffening and cardiovascular disease. Metabolism.

[16] Boutouyrie P, Chowienczyk P, Humphrey JD, Mitchell GF (2021). Arterial stiffness and cardiovascular risk in hypertension. Circ Res.

[17] Chirinos JA, Segers P, Hughes T, Townsend R (2019). Large-artery stiffness in health and disease: JACC state-of-the-art review. J Am Coll Cardiol.

[18] Alsharari R, Lip GY, Shantsila A (2020). Assessment of arterial stiffness in patients with resistant hypertension: Additional insights into the pathophysiology of this condition?. Am J Hypertens.

[19] Khosravi M, Poursaleh A, Ghasempour G, Farhad S, Najafi M (2019). The effects of oxidative stress on the development of atherosclerosis. Biol Chem.

[20] Petrucci G, Rizzi A, Hatem D, Tosti G, Rocca B, Pitocco D (2022). Role of oxidative stress in the pathogenesis of atherothrombotic diseases. Antioxidants (Basel).

[21] Lechner K, von Schacky C, McKenzie AL (2020). Lifestyle factors and high-risk atherosclerosis: pathways and mechanisms beyond traditional risk factors. Eur J Prev Cardiol.

[22] Caiati C, Stanca A, Lepera ME (2023). Free radicals and obesity-related chronic inflammation contrasted by antioxidants: a new perspective in coronary artery disease. Metabolites.

[23] St Paul A, Corbett CB, Okune R, Autieri MV (2020). Angiotensin II, hypercholesterolemia, and vascular smooth muscle cells: a perfect trio for vascular pathology. Int J Mol Sci.

[24] Ji Q, Chai S, Zhang R, Li J, Zheng Y, Rajpathak S (2024). Prevalence and co-prevalence of comorbidities among Chinese adult patients with type 2 diabetes mellitus: a cross-sectional, multicenter, retrospective, observational study based on 3B study database. Front Endocrinol (Lausanne).

[25] Jain RB (2020). Impact of the co-occurrence of obesity with diabetes, anemia, hypertension, and albuminuria on concentrations of selected perfluoroalkyl acids. Environ Pollut.

[26] Cleven L, Krell-Roesch J, Nigg CR, Woll A (2020). The association between physical activity with incident obesity, coronary heart disease, diabetes and hypertension in adults: a systematic review of longitudinal studies published after 2012. BMC Public Health.

[27] Chandrabose M, Rachele JN, Gunn L (2019). Built environment and cardio-metabolic health: systematic review and meta-analysis of longitudinal studies. Obes Rev.

[28] Dayimu A, Wang C, Li J, Fan B, Ji X, Zhang T, Xue F (2019). Trajectories of lipids profile and incident cardiovascular disease risk: a longitudinal cohort study. J Am Heart Assoc.

[29] Torky A, Sinaii N, Jha S, Desai J, El-Maouche D, Mallappa A, Merke DP (2021). Cardiovascular disease risk factors and metabolic morbidity in a longitudinal study of congenital adrenal hyperplasia. J Clin Endocrinol Metab.

[30] Abera MA, Gebregziabher T, Tesfay H, Alemayehu M (2019). Factors associated with the occurrence of hypertension and dyslipidemia among diabetic patients attending the diabetes clinic of Ayder Comprehensive Specialized Hospital, Mekelle, Ethiopia. East Afr J Health Sci.

[31] Bitencourt FV, Nascimento GG, Costa SA, Andersen A, Sandbæk A, Leite FR (2023). Co-occurrence of periodontitis and diabetes-related complications. J Dent Res.

[32] Clark D 3rd, Colantonio LD, Min YI (2019). Population-attributable risk for cardiovascular disease associated with hypertension in Black adults. JAMA Cardiol.

[33] Lindh M, Banefelt J, Fox KM (2019). Cardiovascular event rates in a high atherosclerotic cardiovascular disease risk population: estimates from Swedish population-based register data. Eur Heart J Qual Care Clin Outcomes.

[34] Alieva AS, Olmastroni E, Reutova OV (2020). Prevalence and relationship between metabolic syndrome and risk of cardiovascular disease: evidence from two population-based studies. Atheroscler Suppl.

[35] Irawati S, Wasir R, Floriaan Schmidt A (2019). Long-term incidence and risk factors of cardiovascular events in Asian populations: systematic review and meta-analysis of population-based cohort studies. Curr Med Res Opin.

[36] Rabi DM, McBrien KA, Sapir-Pichhadze R (2020). Hypertension Canada’s 2020 comprehensive guidelines for the prevention, diagnosis, risk assessment, and treatment of hypertension in adults and children. Can J Cardiol.

[37] Litwin M, Kułaga Z (2021). Obesity, metabolic syndrome, and primary hypertension. Pediatr Nephrol.

[38] Elkins C, Fruh S, Jones L, Bydalek K (2019). Clinical practice recommendations for pediatric dyslipidemia. J Pediatr Health Care.

[39] Alonso R, Perez de Isla L, Muñiz-Grijalvo O, Mata P (2020). Barriers to early diagnosis and treatment of familial hypercholesterolemia: current perspectives on improving patient care. Vasc Health Risk Manag.

[40] Christian Flemming GM, Bussler S, Körner A, Kiess W (2020). Definition and early diagnosis of metabolic syndrome in children. J Pediatr Endocrinol Metab.

[41] Belardo D, Michos ED, Blankstein R (2022). Practical, evidence-based approaches to nutritional modifications to reduce atherosclerotic cardiovascular disease: An American Society for Preventive Cardiology Clinical Practice statement. Am J Prev Cardiol.

[42] Barone Gibbs B, Hivert MF, Jerome GJ (2021). Physical activity as a critical component of first-line treatment for elevated blood pressure or cholesterol: who, what, and how?: A scientific statement from the American Heart Association. Hypertension.

[43] Rippe JM (2019). Lifestyle strategies for risk factor reduction, prevention, and treatment of cardiovascular disease. Am J Lifestyle Med.

[44] Carey RM, Moran AE, Whelton PK (2022). Treatment of hypertension: a review. JAMA.

[45] Korhonen MJ, Pentti J, Hartikainen J (2020). Lifestyle changes in relation to initiation of antihypertensive and lipid‐lowering medication: a cohort study. J Am Heart Assoc.

[46] Lip GY, Lane DA, Lenarczyk R (2022). Integrated care for optimizing the management of stroke and associated heart disease: a position paper of the European Society of Cardiology Council on Stroke. Eur Heart J.

[47] Buckley BJ, Lip GY (2022). Current concepts: comprehensive “cardiovascular health” rehabilitation—an integrated approach to improve secondary prevention and rehabilitation of cardiovascular diseases. Thromb Haemost.

[48] Giallauria F, Strisciuglio T, Cuomo G (2021). Exercise training: the holistic approach in cardiovascular prevention. High Blood Press Cardiovasc Prev.

[49] Commodore-Mensah Y, Loustalot F, Himmelfarb CD (2022). Proceedings from a National Heart, Lung, and Blood Institute and the Centers for Disease Control and Prevention workshop to control hypertension. Am J Hypertens.

[50] Roger VL, Sidney S, Fairchild AL (2020). Recommendations for cardiovascular health and disease surveillance for 2030 and beyond: a policy statement from the American Heart Association. Circulation.

[51] Mendis S, Graham I, Narula J (2022). Addressing the global burden of cardiovascular diseases: need for scalable and sustainable frameworks. Glob Heart.

[52] Hirashiki A, Shimizu A, Nomoto K, Kokubo M, Suzuki N, Arai H (2022). Systematic review of the effectiveness of community intervention and health promotion programs for the prevention of non-communicable diseases in Japan and other East and Southeast Asian countries. Circ Rep.

[53] Ndejjo R, Hassen HY, Wanyenze RK (2021). Community-based interventions for cardiovascular disease prevention in low-and middle-income countries: a systematic review. Public Health Rev.

[54] Soltani S, Saraf-Bank S, Basirat R (2021). Community-based cardiovascular disease prevention programmes and cardiovascular risk factors: a systematic review and meta-analysis. Public Health.

